# Assessment of Insertion Sequence Mobilization as an Adaptive Response to Oxidative Stress in Acinetobacter baumannii Using IS-seq

**DOI:** 10.1128/JB.00833-16

**Published:** 2017-04-11

**Authors:** Meredith S. Wright, Stephanie Mountain, Karen Beeri, Mark D. Adams

**Affiliations:** J. Craig Venter Institute, La Jolla, California, USA; University of Tennessee

**Keywords:** Acinetobacter, insertion sequence, mobile genetic elements

## Abstract

Insertion sequence (IS) elements are found throughout bacterial genomes and contribute to genome variation by interrupting genes or altering gene expression. Few of the more than 30 IS elements described in Acinetobacter baumannii have been characterized for transposition activity or expression effects. A targeted sequencing method, IS-seq, was developed to efficiently map the locations of new insertion events in A. baumannii genomes and was used to identify novel IS sites following growth in the presence of hydrogen peroxide, which causes oxidative stress. Serial subculture in the presence of subinhibitory concentrations of hydrogen peroxide led to rapid selection of cells carrying an IS*Aba1* element upstream of the catalase-peroxidase gene *katG*. Several additional sites for the elements IS*Aba1*, IS*Aba13*, IS*Aba25*, IS*Aba26*, and IS*Aba125* were found at low abundance after serial subculture, indicating that each element is active and contributes to genetic variation that may be subject to selection. Following hydrogen peroxide exposure, rapid changes in gene expression were observed in genes related to iron homeostasis. The IS insertions adjacent to *katG* resulted in more than 20-fold overexpression of the gene and increased hydrogen peroxide tolerance.

**IMPORTANCE** Insertion sequences (IS) contribute to genomic and phenotypic variation in many bacterial species, but little is known about how transposition rates vary among elements or how selective pressure influences this process. A new method for identifying new insertion locations that arise under experimental growth conditions in the genome, termed IS-seq, was developed and tested with cells grown in the presence of hydrogen peroxide, which causes oxidative stress. Gene expression changes in response to hydrogen peroxide exposure are similar to those observed in other species and include genes that control free iron concentrations. New IS insertions adjacent to a gene encoding a catalase enzyme confirm that IS elements can rapidly contribute to adaptive variation in the presence of selection.

## INTRODUCTION

Insertion sequences are a significant contributor to genetic change in bacterial genomes. We recently surveyed the distribution of IS elements in sequenced Acinetobacter baumannii genomes and found considerable variations in insertion site location and the abundance of different elements across the phylogeny (1). More than 5,000 different insertion sites for 29 IS elements were found across 976 A. baumannii genomes. Phylogenetically close strains had similar patterns of IS element locations, and the degree of site sharing decreased with phylogenetic distance, indicating that many sites remain stable over long periods of time. However, we also observed new distinct insertion sites for IS elements that arose since strain divergence in very closely related isolates, including in sets of strains obtained from individual patients over a period of days (2), that can have significant impacts on transcription (3). This suggests that IS elements can actively mobilize and may contribute to genome variation over short time intervals.

IS elements have played extensive roles in bacterial adaptation to antibiotic selective pressures. Several IS elements, including IS*Aba1*, which is found upstream of the chromosomal β-lactamase gene *bla*_ADC_ in many strains, are associated with antibiotic resistance markers in A. baumannii. Insertion of IS*Aba1* at that site leads to overexpression of the Acinetobacter-derived cephalosporinase (ADC) protein and resistance to third-generation cephalosporins, including ceftriaxone and cefotaxime (4–6). Pairs of IS elements can act as composite transposons, mobilizing resistance or other genes, including *bla*_OXA-235_, which is flanked by inverted-repeat copies of IS*Aba1* (7).

Few IS elements other than IS*Aba1* have been characterized in A. baumannii in detail. IS*Aba125* is present in many strains in the context of Tn*aphA6*. This composite transposon encodes a 3′-aminoglycoside phosphotransferase type VI that confers amikacin resistance and is flanked by direct-repeat copies of IS*Aba125* (8, 9). IS*Aba125* can cause overexpression of the chromosomal beta-lactamases in A. baumannii (10). It is also the primary means of dissemination of the *bla*_NDM_ beta-lactamase in A. baumannii (11, 12). Although IS*Aba13*, IS*Aba25*, and IS*Aba26* have been described in only a few strains, including LAC-4 (13, 14), each is present in several hundred A. baumannii genomes (1), and nothing is known about their transposition frequency or ability to impact the expression of adjacent genes.

We were interested in further exploring the potential of IS element mobilization to confer a selective advantage to A. baumannii. The LAC-4 strain was selected for study because it contains multiple copies of five different IS elements, namely, IS*Aba1* (21 copies), IS*Aba13* (24 copies), IS*Aba25* (16 copies), IS*Aba26* (14 copies), and IS*Aba125* (16 copies). The selective condition chosen for study was growth in the presence of hydrogen peroxide (H_2_O_2_). H_2_O_2_ breakdown produces hydroxyl radicals that have been shown to mediate bactericidal activity of multiple classes of antibiotics (15). In mammals, neutrophils produce H_2_O_2_ as part of the cytotoxic activity of phagosomes (16). Bacteria exhibit a multifaceted response to this oxidative stress (17). To facilitate identification of new IS locations after growth under stress conditions, we adapted a method that has been used to map the insertion locations of Tn*5* in a transposon library (18). This IS-seq method provides high sensitivity for detecting new insertions, including those at low abundance in a cell population.

## RESULTS

### IS-seq analyses.

**FIG 1:**
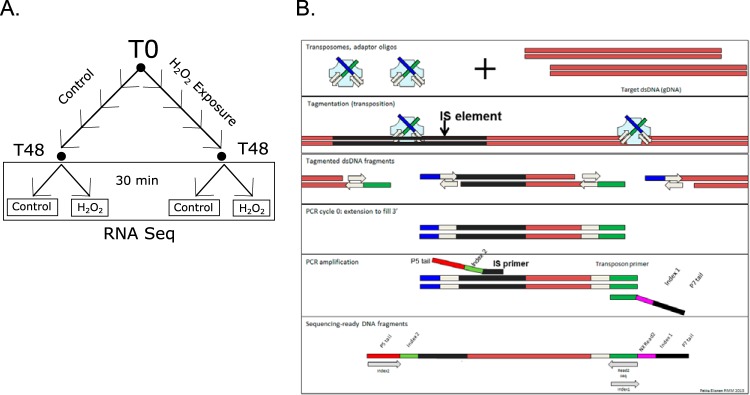
Design of the study. (A) Cell culture scheme illustrating control and H_2_O_2_-treated lineages in which subcultures in fresh LB and fresh H_2_O_2_ were performed every 8 h. After five subculture growths (48 h [T48]), cells were split into control and treatment groups, and the treatment groups were exposed to 8 mM H_2_O_2_ for 30 min. IS-seq analysis was performed on genomic DNA isolated from T0 and T48 cultures. The experiment was performed with three replicate lineages. (B) IS-seq strategy. Illumina Nextera XT tagmentation reaction mixtures were amplified using one custom IS-specific primer with a P5 adapter tail and a standard index-tagged P7 primer. The schematic was adapted from figures originally designed by Illumina and by Pekka Ellonen and used with permission.

The locations of IS*Aba1*, IS*Aba13*, IS*Aba25*, IS*Aba26*, and IS*Aba125* in three replicate cultures (R1, R2, and R3) were determined by IS-seq following growth in the presence or absence of hydrogen peroxide (Fig. 1). All known locations for each element were identified with similar representations in the IS-seq data (see Fig. S1 in the supplemental material). Two composite transposons comprising IS elements flanking a resistance gene (the *bla*_OXA-235_ gene flanked by inverted copies of IS*Aba1*), termed Tn*6252* (14) and Tn*aphA6* (8), are present in two copies each in the LAC-4-jcvi genome. The internal flanks of both composite transposons are represented by approximately twice the IS-seq read density as other chromosomal flanks, demonstrating the utility of read counts for quantifying IS insertions.

**FIG 2:**
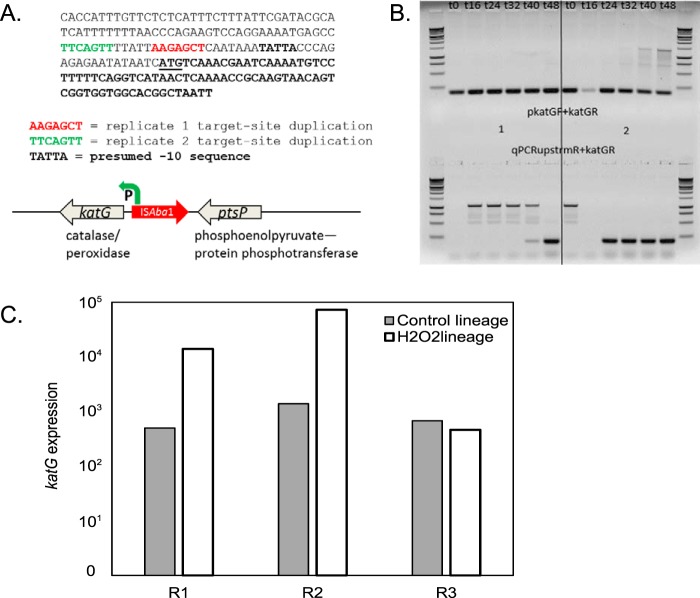
Catalase expression is elevated due to a promoter insertion of IS*Aba*1 in *katG*. (A) *katG* promoter region schematic. (B) PCR assay for detection of IS*Aba1* upstream of *katG* in replicates 1 and 2. The upper band in amplifications using qPCRupstreamR and katGR represents the product of single-primer amplification of the Tn*6252* cassette that is flanked by inverted-repeat copies of IS*Aba1*. (C) Transcript levels of *katG*, expressed as normalized read counts from DESeq2.

Novel insertion sites were also detected based on the analysis of sequences flanking the edges of IS elements. In the T48 samples (final cultures at 48 h) of cells grown in the presence of hydrogen peroxide, a large number of reads supported distinct insertion events of IS*Aba1* elements upstream of the *katG* gene in replicates R1 and R2 (Fig. 2A). The KatG protein is a bifunctional hydroperoxidase I (HPI), exhibiting both catalase and peroxidase activity involved in detoxifying hydrogen peroxide (19), and was shown to be the major contributor to H_2_O_2_ resistance in Acinetobacter species (25). Based on the percentage of reads in each IS-seq amplification pool at the *katG* sites, less than half of the cells contained the IS*Aba1-katG* insertion in R1, while most cells in R2 contained the insertion. Based on read coverage of the whole-genome sequence libraries generated from the T48 whole-genome sequencing (WGS) data, 40% and 80% of cells carry the IS*Aba1* insertions in R1 and R2, respectively. We used PCR amplification to determine when during the serial subculture the IS*Aba1* insertions occurred upstream of *katG*. PCR primers were designed to amplify the *katG* promoter region both with and without the IS element (Table 1). As shown in Fig. 2B, the IS*Aba1* element was detected in R2 at the second postexposure time point (*T* = 16 h). In contrast, the element was apparent at only T40 and T48 in R1. In both cases, the wild-type sequence was also represented at all time points. Although the assay was not quantitative, the proportion of T48 cells carrying IS*Aba1* upstream of *katG* in R1 appeared lower than that in R2 by PCR, which is consistent with data from whole-genome sequencing and IS-seq analysis. Assembled reads also support the idea that the majority of R2 cells carried this insertion, as an IS*Aba1* copy was present in the genome assembly of this sample. Expression of *katG* was also 5-fold higher in R2 than in R1.

**TABLE 1:** Primers used for IS-seq amplification and *katG* insertion testing

Name	Sequence	Purpose
IS*Aba1*-DP	AATGATACGGCGACCACCGAGATCTACACTCTTTCCCTACACGACGCTCTTCCGATCTNNNNNNGAGTTTAAGGAAATTTTGGCAATTTTAAGAARGC	IS-specific primer for amplification of flanking sequences
IS*Aba1*-UP	AATGATACGGCGACCACCGAGATCTACACTCTTTCCCTACACGACGCTCTTCCGATCTNNNNNNGATGTGTCATAGTATTCGTCGTTAG	IS-specific primer for amplification of flanking sequences
IS*Aba125*-DP	AATGATACGGCGACCACCGAGATCTACACTCTTTCCCTACACGACGCTCTTCCGATCTNNNNNNCAAACTGTCGCACCTCATG	IS-specific primer for amplification of flanking sequences
IS*Aba125*-UP	AATGATACGGCGACCACCGAGATCTACACTCTTTCCCTACACGACGCTCTTCCGATCTNNNNNNGTTTATGTCGCACTTCAAGTTTTAC	IS-specific primer for amplification of flanking sequences
IS*Aba13*-DP	AATGATACGGCGACCACCGAGATCTACACTCTTTCCCTACACGACGCTCTTCCGATCTNNNNNNCCACATACCCGAGTTGTCAC	IS-specific primer for amplification of flanking sequences
IS*Aba13*-UP	AATGATACGGCGACCACCGAGATCTACACTCTTTCCCTACACGACGCTCTTCCGATCTNNNNNNGGAATAAGCCTTTAGAGATAGGTTTG	IS-specific primer for amplification of flanking sequences
IS*Aba25*-DP	AATGATACGGCGACCACCGAGATCTACACTCTTTCCCTACACGACGCTCTTCCGATCTNNNNNCACTGCTGGAAACCTAAATCG	IS-specific primer for amplification of flanking sequences
IS*Aba25*-UP	AATGATACGGCGACCACCGAGATCTACACTCTTTCCCTACACGACGCTCTTCCGATCTNNNNNGCCTATAGGATCGCTTGGTAAG	IS-specific primer for amplification of flanking sequences
IS*Aba26*-DP	AATGATACGGCGACCACCGAGATCTACACTCTTTCCCTACACGACGCTCTTCCGATCTNNNNNGTACCTTTTGTAATTATCCTCTGAAGG	IS-specific primer for amplification of flanking sequences
IS*Aba26*-UP	AATGATACGGCGACCACCGAGATCTACACTCTTTCCCTACACGACGCTCTTCCGATCTNNNNNCAGTTCGATGATCGATTAAAAGATC	IS-specific primer for amplification of flanking sequences
P7	CAAGCAGAAGACGGCATACGAGATnnnnnnGTCTCGTGGGCTCGG^*a*^	Standard Illumina primer for IS-seq amplification
kat*G*F	CACCATTTGTTCTCTCATTTCTTTATTCG	Test for IS*Aba1* adjacent to *katG*
kat*G*R	CCACCACCGACTGTTACTTG	Test for IS*Aba1* adjacent to *katG*
qPCRupstreamR	CATTGAGATGTGTCATAGTATTCGTCGTTAG	Test for IS*Aba1* adjacent to *katG*

a The n region comprises the index tag.

Several novel IS insertion sites were also present at very low abundance. Twenty-two novel sites were found across the cultures, each present at <0.03% of the amplification pool (see Table S1). Each of these sites was supported by reads from both upstream and downstream IS edges and showed evidence of target site duplication. Ten of these novel sites were seen in a single replicate and time point, while the other 12 were observed in two or more samples. The novel IS*Aba13* site at coordinate 1729585 was seen in six of the nine assayed samples, including all three of the T0 replicates, suggesting that it was present in the starting culture and maintained in a small subset of cells throughout the experiment.

### RNA-seq results.

**FIG 3:**
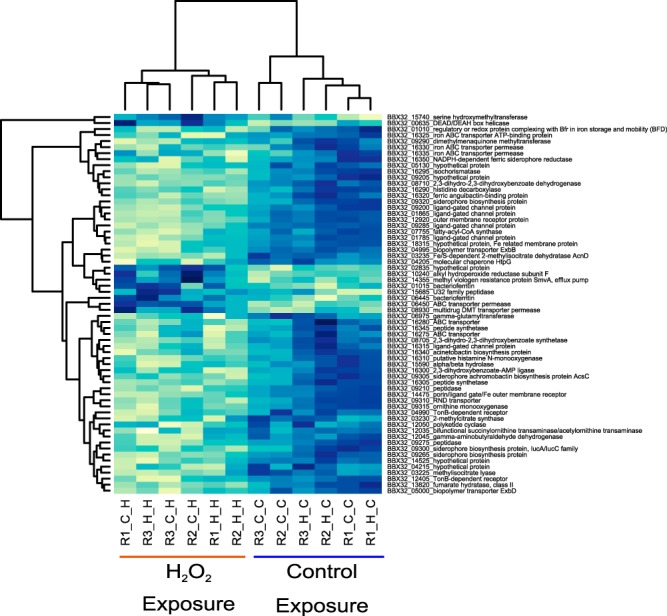
Heat map of differentially expressed genes. Expression levels of the 64 differentially expressed genes are shown as the variance-stabilizing transformed (VST) read count data from DESeq2 (18). Each sample is named with the replicate number, the serial passage subculture condition, and the 30-min exposure condition. For example, R1_H_H indicates replicate 1 grown in H_2_O_2_-amended LB and treated with a brief H_2_O_2_ exposure.

Other than *katG* (discussed below), there were no genes differentially expressed between the H_2_O_2_-adapted and nonadapted samples in the control pulse exposures. Both control and H_2_O_2_-treated lineages also responded similarly to the H_2_O_2_ pulse in all three replicates, resulting in 64 differentially expressed genes in the H_2_O_2_-exposed cells (Fig. 3; see also Table S2 in the supplemental material). In H_2_O_2_-treated cells, genes related to iron metabolism were the most significantly differentially expressed. Expression of genes related to iron transport and acquisition were downregulated, while expression of bacterioferritin, an iron-sequestering protein, was strongly upregulated. Iron contributes to generation of hydroxyl radicals via the Fenton reaction, and iron homeostasis is known to be tightly linked to oxidative stress (21, 22).

Expression of the *katG* gene varied between the control and H_2_O_2_ lineages regardless of pulse experimental conditions in the two T48 replicates with upstream IS*Aba1* insertions. In these two T48 replicates, *katG* was one of the most highly expressed genes (Fig. 2C). Normalized read counts for *katG* were 50 and 150 times higher in replicates 1 and 2, respectively, than in the control cells grown in the absence of H_2_O_2_.

### IS variation and expression.

In the PacBio assembly of LAC-4-jcvi, all copies of IS*Aba13*, IS*Aba125*, IS*Aba25*, and IS*Aba26* are identical to each other. Four versions of the IS*Aba1* element are present; 15 of the 20 copies are identical to one another. Three IS*Aba1* copies share six variant positions and the remaining two copies have three or four of the same variants. One of the variant positions was close enough to the end of the IS that it was decoded as part of the IS-seq amplicons. Both *katG* IS*Aba1* insertions were due to insertion of the major variant of LAC-4-jcvi IS*Aba1*. The RNA-seq data were used to the determine expression level of each IS element relative to its genomic copy number. There were no differences in IS expression levels in any of the growth conditions (data not shown). IS elements differed in expression by about 10-fold, with IS*Aba125* being most highly expressed and IS*Aba1* expressed at the lowest level (Table 2).

**TABLE 2:** Expression levels of IS elements

IS element	Element length (bp)	RPKM per copy (mean ± SD)^*a*^
IS*Aba1*	1,186	1.85 ± 0.69
IS*Aba25*	2,491	6.34 ± 2.31
IS*Aba26*	1,318	11.3 ± 5.58
IS*Aba13*	1,039	12.4 ± 3.03
IS*Aba125*	1,087	18.0 ± 6.88

a RPKM (reads per kilobase per million) per copy is the number of RNA-seq reads divided by the IS element length times the number of copies in the genome times one million. SD, standard deviation.

## DISCUSSION

The IS-seq method detected two independent insertion events upstream of *katG* in response to H_2_O_2_ exposure, which resulted in increased expression of the gene. The concentration of H_2_O_2_ used was chosen to be below the level that would impact the growth rate. Prior growth in H_2_O_2_, with subculture in fresh medium with or without H_2_O_2_ every 8 h, resulted in no changes in gene expression other than in the replicates that had acquired the IS*Aba1* element upstream of *katG*. In contrast, numerous transcriptional changes were identified following a 30-min exposure of T48 cells to H_2_O_2_ regardless of prior exposure, suggesting that regulatory changes provided an adequate adaptation to H_2_O_2_ exposure. *katG* overexpression in replicates 1 and 2 (due to the insertion of IS*Aba1* in the promoter region) represents an additional level of response. The *katG* gene was not overexpressed in replicate 3, implying that the IS insertion events were not essential for providing resistance to the oxidative stress under the sub-MIC growth conditions used here. However, broth microdilution assays of the T48 cultures showed that replicate R1 had a 2-fold increase and replicate R2 had a 4-fold increase in the MIC for H_2_O_2_ compared with replicate R3 and the T0 cultures (data not shown). Variant detection analysis from the T48 WGS data did not reveal any *de novo* mutations in any of the replicates.

In the presence of metal ions, H_2_O_2_ can decompose into the free radicals HO· and HOO·, which are highly reactive and can be toxic. In fact, H_2_O_2_ has been studied as an environmental decontaminant to stop the spread of multidrug-resistant A. baumannii in acute care settings (23, 24). All bacteria have evolved mechanisms of managing hydroxyl radicals, including reduction of the free metal ion concentration and induction of detoxifying enzymes (21). The significant differences in expression of iron acquisition genes seen here are consistent with those previously observed in A. baumannii (25) and other bacteria (26) and provide evidence for coordinated expression of genes related to iron homeostasis.

During development of the IS-seq method, an initial trial was performed using traditional Illumina libraries made by shearing genomic DNA and ligating on standard Illumina adapters. These libraries were then amplified using the Illumina P7 adapter and a custom IS-specific primer tailed with the Illumina P5 adapter sequence. After being sequenced, these libraries contained between 10% and 90% genomic fragments that did not contain IS-flanking sequences, presumably because specific amplification was not adequate for overcoming the large number of adapter-ligated fragments of appropriate size in the starting library. We then switched to using the tagmentation reaction from Nextera XT libraries, followed by amplification. The resulting sequence reads from the amplification products of these libraries were essentially all (>99%) derived from IS-specific priming.

A potential limitation of the IS-seq method presented here is that more cycles of amplification (18 cycles) were required to obtain adequate material for sequencing than when using libraries made using adapter ligation. Additional cycles of PCR might skew the representation of IS-flanking sequences, so relative abundances, particularly of low-abundance novel sites, should be viewed as approximate. Almost all the low-abundance novel IS insertion sites were IS*Aba13* elements (16 of 22). This could imply that IS*Aba13* is more active than the other elements under the tested growth conditions, but formal determination of transposition rates for each element will require additional work.

IS*Aba1* and IS*Aba125* carry strong promoters (10), but the promoter activities of the other elements have not been characterized. We examined the expression levels of genes adjacent to the known IS*Aba13*, IS*Aba25*, and IS*Aba26* elements using the RNA-seq data. Although the sample size was small, no evidence that these elements confer high levels of expression on adjacent genes was found.

Self-mobilizing IS elements have the potential to create significant genome variation with functional consequences. In a long-term evolutionary study of Escherichia coli, mobilization of IS*5* contributed to growth advantages (27). In A. baumannii, up to ∼3% of the genome can consist of IS elements, representing more than 100 IS element copies, although more typically less than 1% of the genome consists of IS sequences. IS mobilization is likely to be important in a clinical context as well. Figueiredo et al. described the upstream insertion of IS*Aba1* and upregulation of the *bla*_OXA-66_ gene following treatment with imipenem in A. baumannii clinical isolates (28). Detailed analysis of Tn*125* carrying the IS*Aba125*-flanked *bla*_NDM_ gene demonstrated mobilization of the transposon during growth but no influence of temperature or subinhibitory antibiotic concentration on the transposition rate (29). The selective forces that regulate the mobilization of IS elements or present limits on their spread are not well known (30). The method presented here is an efficient and low-cost way to determine IS locations in a mixed cellular population and should be of value in further characterizations of the transposition potential of IS elements in diverse bacterial species.

## MATERIALS AND METHODS

### Cell growth.

A. baumannii strain LAC-4 (14) was grown in Luria broth (LB). The MIC of H_2_O_2_ was determined to be 16 mM based on a serial dilution series. Three replicate cultures (R1, R2, and R3) were initiated from single colonies. Overnight cultures of these isolates were transferred to fresh medium and grown to mid-log phase (T0). Each replicate was then split into two subcultures that were grown either without H_2_O_2_ or in the presence of a one-half-concentration MIC (8 mM) added fresh with each passage. Each replicate culture and each condition were passaged every 8 h. An aliquot from each was reinoculated into fresh medium with or without 8 mM H_2_O_2_ with an initial optical density at 600 nm (OD_600_) of 0.05. The final cultures at 48 h (T48) were preserved for genome and transcriptome analysis. An outline of the experimental scheme is shown in Fig. 1.

### IS-seq analysis.

We adapted an approach previously used for mapping the location of transposon insertions (18) for identification of IS-genome junctions. The tagmentation reaction component of the Illumina Nextera XT library preparation method was used to insert Illumina adapter sequences at random locations throughout the genomic DNA (31). The resulting tagged DNA fragments were amplified using the standard barcoded Illumina P7 adapter and a custom primer containing the Illumina P5 adapter, a random nucleotide spacer, and a homology region to the upstream or downstream edge of each IS element (Table 1). By performing PCR amplification from tagmentation reactions using an IS-specific primer tailed with the Illumina P5 sequence along with a standard barcoded P7 amplification primer, we achieved nearly 100% efficiency in obtaining IS-flank junction reads from the whole-genome libraries. We performed IS-seq on the T0 and T48 cultures from each replicate series (R1, R2, and R3) from both the adapted and nonadapted lineages for a total of nine growth conditions. Separate amplification primers were designed to amplify IS-genome junction fragments and enable sequencing from the upstream and downstream edges of each IS element into the adjacent flanking sequence (Table 1). Altogether, 90 amplification products, representing five IS elements, two flanking edges for each element, and nine growth conditions, were combined for Illumina sequencing. With more than 400,000 sequence reads per amplification library, we could quantitate the presence of known and novel IS element insertion sites to a minimum detection limit for novel sites of about 0.01% (∼40 reads supporting each junction).

The resulting IS-seq sequence data were processed using standard Unix commands to produce a set of 40-bp flanking sequences adjacent to the relevant IS element terminus; these were then collapsed into a unique set of sequences, preserving the abundance of each in the library, and compared to the LAC-4 genome by BLAST analysis. The results were stored in a custom MySQL database to facilitate counting of reads supporting each known IS element location and the identification of novel insertion sites. Candidate novel sites were required to be represented by at least 0.002% of reads, to be supported by reads from both IS edges, and to have evidence of the characteristic target site duplication associated with IS insertion.

### Transcriptional response to H_2_O_2_ exposure.

After the final time point, T48 cells were used to inoculate fresh LB, grown to mid-log phase, and then treated with a brief 30-min pulse exposure of 8 mM H_2_O_2_ (treated) or control (untreated) (Fig. 1). These short-term treated and untreated cells were then harvested for RNA-seq analysis.

### DNA and RNA isolation and genome sequencing.

Whole-genome sequencing (WGS) and IS-seq analysis were performed on T48 cultures. Genomic DNA was prepared using the Epicentre Gram-positive DNA isolation kit. RNA was prepared using a MagJet RNA isolation kit (Thermo Fisher) with an additional DNase treatment and rRNA depletion using the Ribo-Zero kit from Illumina. RNA-seq libraries were made on the Wafergen Apollo liquid-handling system using PrepX kits. Whole-genome sequences were obtained for the T48 samples by constructing Illumina Nextera XT libraries and sequencing using 2× 150-base reads on an Illumina NextSeq 500 sequencer. After initial experiments, we found that the LAC-4 strain had diverged since its last ancestor with the version reported as GenBank accession number CP007712.1. We therefore performed Pacific Biosciences (PacBio) single-molecule real-time (SMRT) sequencing on the strain used in our laboratory using standard methods and kits from PacBio. This sequence was termed LAC-4-jcvi.

### Genomic and transcriptome analysis.

WGS reads from T48 genome sequencing were mapped to this PacBio genome to detect any new mutations that arose during the experiment using Bowtie 2 (32) and SAMtools mpileup (33) variant detection. RNA-seq reads were mapped to the LAC-4-jcvi genome sequence and annotation using CLC Genomics Workbench, and raw counts of reads aligning to each gene were used as input to the DESeq2 Bioconductor package (20).

### Accession number(s).

The transcriptome-sequencing data sets were deposited in GenBank under BioProject accession PRJNA357077. The PacBio sequences of LAC-4-jcvi were deposited under GenBank accession numbers CP018677 through CP018679.

## Supplementary Material

Supplemental material
