# Supplementary material for: Assessment of Insertion Sequence Mobilization as an Adaptive Response to Oxidative Stress in Acinetobacter baumannii Using IS-seq
Source: J Bacteriol. 2017 Apr 11;199(9):e00833-16. doi: 10.1128/JB.00833-16 (PMC5388817; doi:10.1128/JB.00833-16)
Supplement: Supplemental material [file supp_199_9_e00833-16__index.html]

Assessment of Insertion Sequence Mobilization as an Adaptive Response to Oxidative Stress in Acinetobacter baumannii Using IS-seq — Supplemental material 

# Assessment of Insertion Sequence Mobilization as an Adaptive Response to Oxidative Stress in Acinetobacter baumannii Using IS-seq

## Supplemental material

- Supplemental file 1 -

  Fig. S1, proportion of reads supporting known IS element sites

  Table S1, novel IS insertion locations

  Table S2, list of differentially expressed genes

  PDF, 535K
