# Supplementary material for: Assessment of Insertion Sequence Mobilization as an Adaptive Response to Oxidative Stress in Acinetobacter baumannii Using IS-seq
Source: J Bacteriol. 2017 Apr 11;199(9):e00833-16. doi: 10.1128/JB.00833-16 (PMC5388817; doi:10.1128/JB.00833-16)
Supplement: Supplemental material [file JB.00833-16_zjb999094373s1.pdf]

Supplemental Tables and Figures

Supplemental Figure 1. Proportion of reads supporting known IS element sites

Supplemental Table 1. Novel IS insertion locations

Supplemental Table 2. List of differentially expressed genes

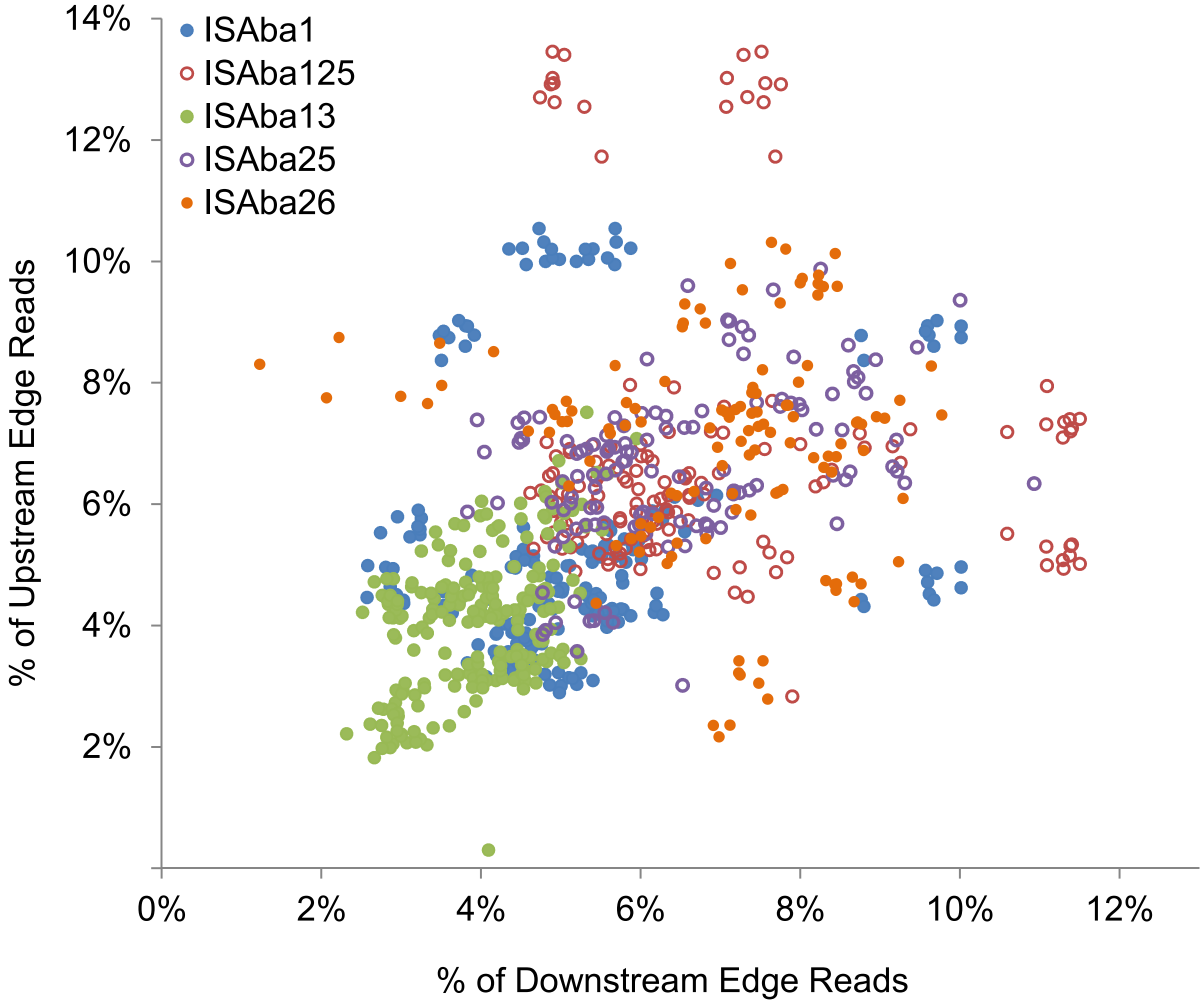

Figure S1. Percentage of IS-seq reads representing each known copy of the five IS elements present in LAC-4-jcvi. Each point represents the genomic location of an IS element copy in the amplification from one of the replicate samples at T0 or T48.

Pair#	is_element	LAC-4-jcvi coordinate	sample	dir	downstream read count	% of downstream reads	dir	upstream read count	% of upstream reads	Notes
1	ISAba13	182	2AT0	D	55	0.005%	U	26	0.004%	
2	ISAba13	222399	1AH2O2T48	D	109	0.026%	U	56	0.008%	between genes
3	ISAba125	304269	1AH2O2T48	D	86	0.012%	U	43	0.006%	in alkene reductase
4	ISAba125	413385	1AT0	D	72	0.005%	U	88	0.004%	in traA conjugal transfer protein
5	ISAba125	1059636	2ACT48	D	54	0.008%	U	25	0.004%	in acetate kinase
6	ISAba13	1337212	1AT0	D	76	0.004%				in trehalose phosphatase
			1ACT48	D	35	0.007%	U	49	0.006%	
7	ISAba13	1729585	2AH2O2T48	D	52	0.010%				3 genes away from adeABC
	ISAba13		3AT0	D	97	0.006%	U	135	0.009%	
	ISAba13		3AH2O2T48	D	63	0.003%				
			2AT0	D	26	0.002%				
			1ACT48				U	81	0.009%	
			1AT0				U	279	0.009%	
8	ISAba13	1822131	1AT0	D	34	0.002%	U	325	0.011%	in alpha-ketoglutarate permease
9	ISAba13	2160935	3AH2O2T48	D	88	0.004%	U	74	0.006%	just upstream of aphA6; inside ISAba125 compound trar
			3ACT48	D	27	0.004%				
10	ISAba13	2348308	2AT0	D	185	0.017%	U	478	0.028%	pyrroline-5-carboxylate reductase
	ISAba13		2ACT48	D	136	0.026%	U	163	0.025%	
11	ISAba13	2693018	3AH2O2T48	D	88	0.004%	U	74	0.006%	just upstream of aphA6; inside ISAba125 compound trar
			3ACT48	D	27	0.004%				
12	ISAba13	2697793	2ACT48	D	70	0.013%	U	83	0.013%	in galactose mutarotase
			2AT0	D	32	0.003%				
13	ISAba13	2900355	3AH2O2T48				U	60	0.005%	no good candidate for this
	ISAba13		3AH2O2T48				U	45	0.004%	
14	ISAba13	2961448	2AT0	D	42	0.004%	U	75	0.004%	intergenic
15	ISAba13	3497793	1AT0	D	145	0.008%	U	32	0.001%	in molecular chaperone
	ISAba13		1ACT48	D	30	0.006%	U	41	0.005%	
16	ISAba13	3501389	1AT0	D	44	0.003%	U	167	0.006%	
17	ISAba13	3501954	3AH2O2T48	D	39	0.002%	U	27	0.002%	
18	ISAba13	3750202	1AT0	D	207	0.012%	U	85	0.003%	intergenic
19	ISAba25	951401	2AT0	D	278	0.042%	U	169	0.026%	in aldehyde-activating protein
	ISAba25		2ACT48	D	420	0.035%	U	300	0.022%	
20	ISAba25	1459662	1ACT48	D	392	0.032%	U	381	0.032%	3' end of crossover junction endodeoxyribonuclease Rus
	ISAba25		1AT0	D	397	0.028%	U	716	0.021%	
21	ISAba25	1541667	3AH2O2T48	D	325	0.024%	U	504	0.040%	intergenic
	ISAba25		3AT0	D	259	0.026%	U	355	0.034%	
	ISAba25							403	0.035%	
22	ISAba25	2766108	3AH2O2T48	D	551	0.041%	U	462	0.037%	in asparagine synthase (glutamine-hydrolyzing)
	ISAba25		3AT0	D	303	0.031%	U	257	0.025%	
	ISAba25		3ACT48				U	583	0.051%	

ısposon

ısposon

;A

Predicted Annotation	LAC4-jcvi Locus Tag	R1 Control Lineage		R1 H2O2 Lineage		R2 Control Lineage		R2 H2O2 Lineage		R3 Control Lineage		R3 H2O2 Lineage	
		Control Exposure	H2O2 Exposure	Control Exposure	H2O2 Exposure	Control Exposure	H2O2 Exposure	Control Exposure	H2O2 Exposure	Control Exposure	H2O2 Exposure	Control Exposure	H2O2 Exposure
DEAD/DEAH box helicase	BBX32_00635	5339.59	37368.84	2358.08	8612.19	1567.32	27034.22	1430.35	2916.67	921.20	5740.67	1346.98	5198.82
ligand-gated channel protein	BBX32_01785	880.77	84.69	928.11	224.21	555.22	128.53	989.18	191.10	367.00	106.62	528.25	60.93
ligand-gated channel protein	BBX32_01865	2047.58	34.69	1715.66	55.44	1304.46	85.69	2252.95	106.90	442.42	26.66	902.30	23.63
hypothetical protein	BBX32_02835	279.93	820.37	310.90	872.18	172.56	1125.27	299.86	334.90	280.80	877.52	337.24	563.28
methylisocitrate lyase	BBX32_03225	193.60	52.04	249.79	66.52	492.64	78.13	431.98	213.81	643.76	62.91	317.35	46.01
2-methylcitrate synthase	BBX32_03230	77.61	15.31	113.82	14.78	272.69	68.05	345.81	162.72	566.32	59.71	391.96	24.87
Fe/S-dependent 2-methylisocitrate dehy	BBX32_03235	629.62	126.53	881.51	139.20	1004.95	199.10	1081.09	363.28	1793.24	161.00	1162.94	171.59
molecular chaperone HtpG	BBX32_04205	688.05	108.16	821.93	336.31	979.91	153.73	769.75	626.29	1197.96	374.25	1592.70	384.22
hypothetical protein	BBX32_04215	170.92	67.34	185.62	68.99	149.31	74.35	269.99	166.50	377.10	113.02	445.68	143.00
TonB-dependent receptor	BBX32_04990	166.56	18.37	179.51	43.12	374.62	70.57	311.35	37.84	70.03	30.92	126.34	14.92
biopolymer transporter ExbB	BBX32_04995	1292.38	162.24	1096.93	351.09	1214.16	432.21	1513.07	506.14	870.02	174.86	1068.43	220.09
biopolymer transporter ExbD	BBX32_05000	463.06	89.79	518.67	167.54	452.40	119.71	366.49	137.18	193.26	57.58	260.64	114.40
hypothetical protein	BBX32_05130	95.05	6.12	67.98	17.25	122.49	13.86	137.87	16.08	21.55	2.13	39.79	2.49
bacterioferritin	BBX32_06445	50.58	579.57	82.50	321.52	67.06	122.23	13.79	107.85	123.23	233.51	40.79	1049.46
ABC transporter permease	BBX32_06450	26.16	101.02	23.68	44.35	24.14	78.13	27.57	36.90	19.53	82.10	16.91	75.85
gamma-glutamyltransferase	BBX32_06975	28.78	12.24	33.61	6.16	88.51	23.94	56.30	27.44	47.14	20.26	68.64	17.41
fatty-acyl-CoA synthase	BBX32_07755	1239.19	117.34	1048.80	325.22	827.92	108.37	889.23	270.57	371.04	60.78	335.25	82.07
2,3-dihydro-2,3-dihydroxybenzoate synt	BBX32_08705	115.98	9.18	160.41	6.16	59.01	16.38	367.64	16.08	82.83	11.73	220.85	7.46
2,3-dihydro-2,3-dihydroxybenzoate dehy	BBX32_08710	52.32	3.06	84.79	6.16	41.13	3.78	167.74	4.73	30.30	4.26	76.60	1.24
multidrug DMT transporter permease	BBX32_08930	61.04	189.79	39.72	105.94	34.87	171.37	67.78	66.22	42.42	73.57	38.80	69.63
ligand-gated channel protein	BBX32_09200	2905.69	32.65	3062.38	82.54	770.70	42.84	1417.72	86.09	282.15	14.93	484.48	23.63
hypothetical protein	BBX32_09205	183.13	3.06	216.94	3.70	41.13	3.78	68.93	4.73	12.12	3.20	30.84	4.97
peptidase	BBX32_09210	435.16	10.20	447.63	22.17	138.58	3.78	268.84	22.71	70.71	10.66	146.24	16.16
siderophore biosynthesis protein	BBX32_09265	620.90	29.59	580.55	45.58	292.36	41.58	1369.46	75.68	238.38	51.18	509.35	21.14
peptidase	BBX32_09275	469.17	47.96	576.73	60.36	129.64	31.50	353.85	63.39	133.33	18.13	213.89	18.65
ligand-gated channel protein	BBX32_09285	2077.23	38.77	2163.30	109.64	1382.25	79.39	2388.52	245.97	1189.21	47.98	1714.07	78.34
dimethylmenaquinone methyltransferas	BBX32_09290	21.80	2.04	12.99	8.62	5.36	7.56	26.42	6.62	10.77	3.20	20.89	3.73
siderophore biosynthesis protein, IucA/I	BBX32_09300	770.90	73.47	563.74	66.52	90.30	84.43	583.63	40.68	90.23	36.25	328.29	80.82
siderophore achromobactin biosynthesis	BBX32_09305	312.20	27.55	228.40	24.64	56.33	27.72	214.84	12.30	41.08	20.26	144.25	37.30
RND transporter	BBX32_09310	593.87	17.35	579.02	23.41	83.15	46.62	526.19	9.46	59.93	23.46	229.80	11.19
ornithine monooxygenase	BBX32_09315	1194.71	15.31	1232.90	20.94	170.77	54.18	965.06	23.65	107.74	24.52	449.66	3.73
siderophore biosynthesis protein	BBX32_09320	2166.18	55.10	2629.26	59.13	226.20	90.73	2233.42	48.25	241.75	45.85	1036.60	36.06
alkyl hydroperoxide reductase subunit F	BBX32_10240	403.76	1209.13	385.76	756.38	360.31	1470.54	411.30	487.22	293.60	844.47	390.96	407.85
arginine N-succinyltransferase	BBX32_12030	162.20	54.08	139.79	89.93	31.29	23.94	68.93	82.31	92.93	31.99	102.47	32.33
bifunctional succinylornithine transamin	BBX32_12035	95.05	33.67	106.18	70.22	55.43	27.72	105.70	69.06	112.46	56.51	92.52	14.92
gamma-aminobutyraldehyde dehydroge	BBX32_12045	159.59	47.96	184.86	81.31	90.30	25.20	91.91	64.33	158.92	29.85	124.35	27.36
polyketide cyclase	BBX32_12050	104.65	82.65	126.80	65.29	87.62	42.84	86.17	71.90	162.29	31.99	192.00	90.77
TonB-dependent receptor	BBX32_12405	539.80	79.59	528.60	158.91	319.19	107.11	363.05	122.99	268.68	30.92	256.66	32.33
outer membrane receptor protein	BBX32_12920	3228.35	43.88	3183.83	62.83	1595.04	81.91	3021.55	85.14	387.87	40.52	1124.14	37.30
fumarate hydratase, class II	BBX32_13820	473.53	68.36	708.11	92.39	291.47	79.39	488.27	70.01	132.66	62.91	278.55	93.26

methyl viologen resistance protein SmvA	BBX32_14355	145.63	394.88	116.87	224.21	128.75	633.83	101.10	140.96	82.15	288.95	166.13	207.65
porin/ligand gate/Fe outer membrane re	BBX32_14475	730.78	6.12	853.25	17.25	218.16	13.86	677.84	30.27	95.62	11.73	200.95	6.22
hypothetical protein	BBX32_14525	443.88	36.73	533.95	73.91	418.43	93.25	900.72	150.42	361.61	67.17	369.08	21.14
alpha/beta hydrolase	BBX32_15590	233.71	27.55	174.93	40.65	84.04	32.76	157.40	26.49	37.04	10.66	70.63	8.70
U32 family peptidase	BBX32_15685	98.54	257.13	99.30	238.99	265.54	317.55	160.84	423.83	318.51	915.90	414.84	1030.81
serine hydroxymethyltransferase	BBX32_15740	2735.63	3920.24	2175.52	4757.58	4754.72	6878.89	3432.85	4331.02	3263.92	5341.89	3115.76	4776.05
ABC transporter	BBX32_16275	87.21	18.37	68.75	8.62	36.66	65.53	272.28	22.71	53.87	40.52	116.39	8.70
ABC transporter	BBX32_16280	75.87	16.33	58.82	18.48	22.35	31.50	198.76	12.30	25.59	14.93	86.55	11.19
histidine decarboxylase	BBX32_16290	80.23	5.10	67.22	1.23	9.83	7.56	91.91	2.84	19.53	5.33	38.80	3.73
isochorismatase	BBX32_16295	90.69	5.10	109.23	6.16	29.50	5.04	78.12	2.84	10.77	3.20	34.82	2.49
2,3-dihydroxybenzoate-AMP ligase	BBX32_16300	235.45	33.67	183.33	6.16	47.39	31.50	271.14	8.51	55.89	29.85	95.50	12.43
peptide synthetase	BBX32_16305	271.21	20.41	273.47	20.94	67.06	35.28	449.21	28.38	63.30	28.79	209.91	22.38
putative histamine N-monooxygenase	BBX32_16310	178.77	19.39	142.84	20.94	35.76	16.38	121.78	17.97	49.16	9.60	93.51	8.70
ligand-gated channel protein	BBX32_16315	169.18	11.22	114.58	9.86	73.31	50.40	405.55	21.76	47.81	28.79	213.89	13.68
ferric anguibactin-binding protein	BBX32_16320	47.09	7.14	30.56	4.93	22.35	6.30	78.12	4.73	14.81	3.20	53.72	6.22
iron ABC transporter ATP-binding protei	BBX32_16325	12.21	2.04	19.86	0.00	5.36	0.00	10.34	4.73	6.73	4.26	9.95	1.24
iron ABC transporter permease	BBX32_16330	34.01	5.10	20.62	7.39	18.78	6.30	42.51	5.68	6.06	11.73	25.87	3.73
iron ABC transporter permease	BBX32_16335	60.17	19.39	45.07	3.70	5.36	12.60	26.42	0.95	6.73	13.86	17.91	3.73
acinetobactin biosynthesis protein	BBX32_16340	270.34	5.10	234.51	19.71	51.86	65.53	390.62	18.92	43.77	24.52	204.93	19.90
peptide synthetase	BBX32_16345	75.00	21.43	133.68	6.16	50.96	39.06	176.93	12.30	41.08	37.32	136.29	13.68
NADPH-dependent ferric siderophore re	BBX32_16350	46.22	14.29	45.07	14.78	19.67	11.34	25.28	6.62	16.16	6.40	23.88	18.65
hypothetical protein, Fe related membra	BBX32_18315	1111.00	104.08	1064.08	305.51	725.99	224.30	1002.97	272.46	493.59	72.50	523.27	59.69
regulatory or redox protein complexing	BBX32_01010	12.21	2.04	28.26	2.46	10.73	1.26	9.19	0.00	0.67	0.00	5.97	0.00
bacterioferritin	BBX32_01015	190.11	573.45	262.01	512.47	168.09	158.77	97.65	210.97	214.81	597.10	150.22	718.71
